# Biological Processes Underlying Genetic Adaptation of Larches to Cold and Dry Winter Conditions in Eastern Siberia

**DOI:** 10.1002/ece3.70940

**Published:** 2025-02-12

**Authors:** Stefano Meucci, Stefan Kruse, Sarah Haupt, Kathleen R. Stoof‐Leichsenring, Konstantin V. Krutovsky, Nadine Bernhardt, Dörte Harpke, Ulrike Herzschuh

**Affiliations:** ^1^ Alfred Wegener Institute Helmholtz Centre for Polar and Marine Research Polar Terrestrial Environmental Systems Potsdam Germany; ^2^ Institute of Biochemistry and Biology University of Potsdam Potsdam Germany; ^3^ Department of Forest Genetics and Forest Tree Breeding George‐August University of Göttingen Göttingen Germany; ^4^ Center for Integrated Breeding Research Georg‐August University of Göttingen Göttingen Germany; ^5^ Laboratory of Population Genetics, N. I. Vavilov Institute of General Genetics Russian Academy of Sciences Moscow Russian Federation; ^6^ Laboratory of Forest Genomics, Genome Research and Education Center, Department of Genomics and Bioinformatics, Institute of Fundamental Biology and Biotechnology Siberian Federal University Krasnoyarsk Russian Federation; ^7^ Scientific and Methodological Center G. F. Morozov Voronezh State University of Forestry and Technologies Voronezh Russian Federation; ^8^ Julius Kühn‐Institut, Federal Research Centre for Cultivated Plants Institute for Resistance Research and Stress Tolerance Quedlinburg Germany; ^9^ Leibniz Institute of Plant Genetics and Crop Plant Research Gatersleben Stadt Seeland Germany; ^10^ Institute of Environmental Science and Geography University of Potsdam Potsdam Germany

**Keywords:** bioclimatic variables, boreal forests, genetic adaptation, genotype–environment associations (GEAs), landscape genomics, larches

## Abstract

The boreal forests of central and eastern Siberia, dominated by larches, are challenged by increasingly harsher continental conditions and more frequent droughts. Despite the crucial ecosystem services provided by these Siberian boreal forests, the major stressors driving the selective factors as well as the genetic adaptation mechanisms of larches are still unknown. Here we present a landscape genomics study on 243 individuals of the dominant larch tree species, *Larix gmelinii* and *L. cajanderi*. We assessed genotype‐environment associations (GEAs) between genetic variation of individual markers based on genotyping‐by‐sequencing (GBS) data and bioclimatic variables recorded at the sampling locations. We find that the cold and dry winter conditions of eastern Siberia are likely the main selective factor driving the genetic adaptation of larches. Gene ontology (GO) enrichment analysis identified metabolic, transmembrane transport, and homeostatic, as well as developmental processes among the main biological processes underlying genetic adaptation driven by cold and dry winter conditions.

## Graphical abstract

We present a landscape genomics study on 243 individuals of the larch tree species *Larix gmelinii* and *L. cajanderi*. We find that the dry, cold conditions of the eastern Siberian winter are likely the main selective factor driving the genetic adaptation of larches. We identified metabolic, transmembrane transport, and homeostatic, as well as developmental processes among the main biological processes underlying genetic adaptation driven by dry, cold winter conditions.

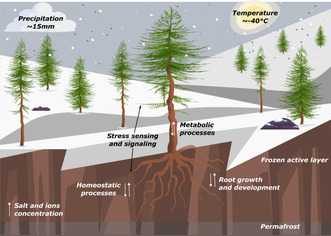

## 1 Introduction

The Siberian forest is the largest forest region on Earth, forming ~60% of all boreal forests, with *Larix gmelinii* (Rupr.) Kuzen and *L. cajanderi* Mayr. as the dominant tree species (Herzschuh 2020; Kayes and Mallik 2020). It provides such crucial ecosystem services as mitigation of climatic fluctuations, permafrost stabilization, and wildlife habitat maintenance (Apps, Shvidenko, and Vaganov 2006; Herzschuh et al. 2016). However, larches are challenged by the environmental conditions of central and eastern Siberia, which are characterized by a continental climate with increasing extremes of winter cold and summer warmth (Abaimov 2010; Sidorova et al. 2021; Miyahara et al. 2011; Ohta et al. 2008; Tchebakova et al. 2023; Wang et al. 2022). Proxy data indicate a general cooling trend that began in the Late Holocene (~4500 BP) until the nineteenth century, particularly in the Northern Hemisphere, north of 60° N (Bader et al. 2020; Kaufman and Broadman 2023). Moreover, an ongoing precipitation decline and permafrost degradation are causing a soil‐water deficit due to evapotranspiration and water infiltration (Sidorova et al. 2021; Miyahara et al. 2011; Ohta et al. 2008; Peng et al. 2022; Tchebakova et al. 2023). Yet the main environmental stressors driving the selective factors and related genetic adaptation mechanisms of eastern Siberian larches are still unknown. Understanding the genetic adaptation mechanisms of Siberian larches is essential for identifying traits that enhance their resilience to environmental stresses. These insights are crucial for predicting the impacts of future environmental changes and for guiding sustainable forest management, including reforestation initiatives and conservation strategies.

Eastern Siberia (e.g., Yakutia) is one of the regions with the greatest seasonal temperature fluctuation in the world, with peaks around −60°C in winter and +35°C in summer. Median precipitation per season is 15 mm in winter and 46 mm in summer (Abaimov 2010). There is evidence that extreme temperatures, drought, and salinity are among the main stressors that can alter the physiology and biochemistry of plants as well as denature their biological structures (Baldi and La Porta 2022; Eckert et al. 2009; Isah 2019; Miller et al. 2020; Zhang et al. 2022). Drought stress increases salt concentration in the soil of larch forests (Basu et al. 2016; Herzschuh et al. 2013), and both drought and salinity induce hyperosmotic stress in plant cells (Yang and Guo 2018; Zhang, Zhao, and Zhu 2020). Larches have an average reproductive phase spanning 20 to 200 years, with *L. gmelinii* and *L*. *cajanderi* recorded to live up to ~600 years in Taimyr and ~ 880 years in the Indigirka River basin, respectively (Abaimov 2010). Therefore, both long‐term and short‐term climate have likely shaped the population's genetic makeup, serving as determinants of *Larix* species adaptation and distribution (Gloy, Herzschuh, and Kruse 2023; Haupt et al. 2024; Schulte et al. 2022). However, since no genetic data are available, it is still an open question as to whether cold and dry winters or increasingly warm and dry summers are the major stressors driving genetic adaptation of larches and their range shifts in eastern Siberia (Mamet et al. 2019; McDowell et al. 2008; Zhirnova et al. 2020).

Physiological adaptation studies on plant‐stress responses show that both heat and cold stress, as well as water loss, can alter the membrane fluidity and cause denaturation of protein and RNA structures, which might induce the arrest of cell growth and photosynthesis (Baldi and La Porta 2022; Isah 2019; Miller et al. 2020; Huiming Zhang et al. 2022). Gene expression studies in conifers show that genes with putative roles in drought‐stress responses are involved in cell‐wall reinforcement, maintenance of osmotic balance, sugar metabolism, and protection of cytoplasmic structures (Eveno et al. 2008). The genetic adaptation of conifers to abiotic stresses has mostly been studied in American and European species of *Pinus*, *Picea*, and *Abies* (Eckert et al. 2009, 2010; Eveno et al. 2008; Lu, Loopstra, and Krutovsky 2019; Prunier et al. 2011, 2012), while from the Siberian boreal forest only the western and southern populations of *L. sibirica* have been studied (Novikova, Oreshkova, et al. 2023, Novikova, Sharov, et al. 2023). The variety of candidate adaptive genes in conifers with putative roles in abiotic‐stress response reveals the complexity of adaptation mechanisms apparently related to species traits and local environments (Howe et al. 2003; Mosca, González‐Martínez, and Neale 2014), but the biological processes underlying genetic adaptation of *L. gmelinii* and *L. cajanderi* to the harsh environmental conditions of eastern Siberia remain unexplored.

Landscape genomics provides tools to investigate genotype–environment associations (GEAs) to explore how genetic variation correlates with bioclimatic variables, thereby uncovering patterns of genetic adaptation to local environmental conditions (Balkenhol et al. 2019; Rellstab et al. 2015). To genotype individual trees, an efficient approach is to reduce genome complexity using genotyping‐by‐sequencing (GBS). This method often employs methylation‐sensitive restriction enzymes to selectively fragment and sequence DNA, avoiding highly methylated repetitive regions. As a result, it enables the genotyping of thousands of single nucleotide polymorphisms (SNPs), with a relatively random and even focus on low‐copy gene‐rich regions of the genome (Ashwath et al. 2023; Elshire et al. 2011; Poland et al. 2012; Wendler et al. 2014). Different strategies using either a single method or a combination of several methods are available to investigate the association between genotypes and environmental variables (De Villemereuil et al. 2014; Joost et al. 2013; Rellstab et al. 2015). Among the GEAs tools, Samβada can perform many logistic regressions to test whether a bioclimatic variable affects the allele and genotype frequencies (Duruz et al. 2019; Stucki et al. 2017). Since the selectively neutral genetic structure due to genetic drift, gene flow, isolation, and mutation might mimic patterns of genetic adaptation, it is recommended that selectively neutral population structure and spatial autocorrelation are accounted for as confounding factors (Rellstab et al. 2015). However, to correct for both selectively neutral genetic and spatial structure might be overly conservative (Vilhjálmsson and Nordborg 2013). Additionally, a gene ontology (GO) enrichment analysis might be implemented to identify whether a certain biological process, molecular function, or cellular component is over‐ or under‐represented in a set of candidate adaptive genes whose variation demonstrates significant associations with environmental factors and bioclimatic variables (Rellstab et al. 2015).

Here we present a GEA study on 243 individual larch trees distributed across a large study area from the north Krasnoyarsk Krai region (Russia) to the Chukotka Autonomous Okrug, or Chukotka (Far East, Russia). We have genotyped the individual trees using GBS and evaluated their population structure. We performed GEA analysis and explored the main stressors driving genetic adaptation of *Larix* in eastern Siberia. Finally, we investigated the biological processes enriched by potential candidate adaptive genes via GO enrichment analysis and semantic similarity clustering.

## 2 Materials and Methods

### 2.1 Study Area, Plant Materials, and Environmental Variables

**FIGURE 1:**
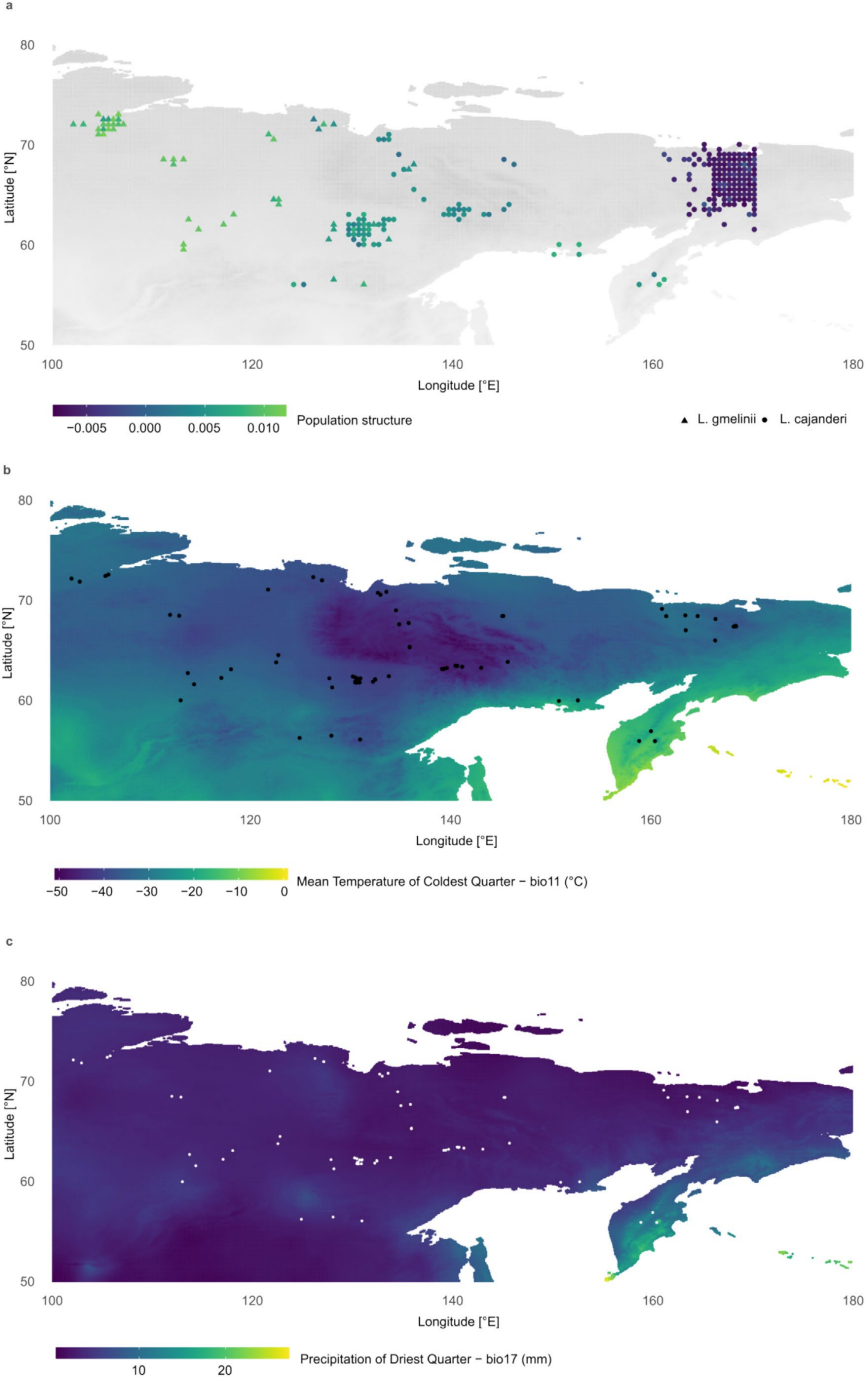
(a) Map of the study area with triangles representing *L. gmelinii* individuals and circles representing *L. cajanderi* individuals with color reflecting population structure according to the scores of the first PCA component based on the genotype data. The individuals were spread on the map to allow visibility of all samples. (b) Map of the study area with background color representing the WorldClim bioclimatic variable Mean Temperature of Coldest Quarter (Bio11); black dots represent individual sampling sites. (c) Map of the study area with background color representing the WorldClim bioclimatic variable precipitation of driest quarter (Bio17) with white dots representing individual sampling sites.

Needle samples were collected from 243 individual trees, morphologically identified as 196 *L. cajanderi* and 47 *L. gmelinii* . The samples were collected during different summer expeditions between 2011 and 2021 in an area spanning from the Krasnoyarsk Krai region to Chukotka and Kamchatka (Russia), including Arctic and subarctic regions (Figure 1). The needles were dried on silica gel during fieldwork and stored at 4°C until processing. A sample list with detailed information and geographic coordinates is presented in Table S1. Bioclimatic variables (Bio1–Bio19) and historical climate data (precipitation, minimum and maximum temperature) corresponding to the sampling location of the individual trees with 30 s (~1 km^2^) spatial resolutions were downloaded from the WorldClim database v. 2.1 (Fick and Hijmans 2017) (Table S2) using the *createEnv* function integrated into the Samβada landscape genomic tool. Maps (Figure 1) of seasonal and annual distribution (Figure S1) were plotted in R using the package ggplot2 (R Core Team 2022; Wickham 2016). Correlation analysis and hierarchical clustering of bioclimatic variables were done with the R package corrplot v. 0.92 (Wei and Simko 2021) (Figure S2).

### 2.2 DNA Extraction and GBS

The needles (40 to 80 mg) were transferred into impact‐resistant 2 mL tubes together with two DNA‐free steel beads of 5 mm diameter and ground to a powder with FastPrep‐24 (MP Biomedicals, USA) for 50 s at 4 m s^−1^. The Invisorb Spin Plant Mini Kit (Stratec Molecular, Germany), DNeasy Plant Mini Kit (Qiagen, Germany) or DNeasy Plant Pro Kit (Qiagen, Germany), were used to extract total genomic DNA following the manufacturer protocol.

GBS library preparation and sequencing were performed following the protocol of Wendler et al. (2014). Genomic DNA was digested using restriction enzymes *Pst*I and *Msp*I. Libraries were sequenced on an Illumina HiSeq 2000 and Illumina NovaSeq 6000, generating single‐end reads of 100 bp. Raw reads were demultiplexed with the CASAVA pipeline 1.8 (Illumina, USA). The number of raw reads produced for each sample is provided in Table S3. The demultiplexed Illumina sequence data have been deposited in the European Nucleotide Archive (ENA) at EMBL‐EBI under accession number PRJEB71740 (https://www.ebi.ac.uk/ena/data/view/PRJEB71740).

### 2.3 GBS Data Assembly and Consensus Base Calling

Raw reads were trimmed, filtered, and *de novo* assembled with ipyrad v. 0.9.84 (Eaton and Overcast 2020). Consensus base calling was integrated into the ipyrad pipeline. Minimum coverage for base calls was set to six per base. At least 90% of sequence similarity was set as the threshold for which two sequences are identified as being homologous and thus cluster together. The minimum number of samples that must have data at a given locus was set to 60% (*min samples per locus* = 146). The maximum heterozygosity per site was not allowed to exceed 0.6 in order to exclude the potential occurrence of stacked paralogues. Further parameters were set to default. The assembled reads had a mean length of ca. 105 bp per locus. A summary of assembled data is provided in Table S3. To explore an alternative approach for filtering potential paralogs, we evaluated the HDplot method (McKinney et al. 2017), which integrates heterozygote proportions with deviations in allele read ratios (Data S1). Implementing HDplot required de novo assembly using the Stacks pipeline (Catchen et al. 2013).

### 2.4 Population Structure, GEA, Heterozygosity, and Spatial Autocorrelation

The cross‐validation procedure and the ancestral components were estimated using ADMIXTURE v1.3 (Alexander, Novembre, and Lange 2009). GEA analysis was performed using the Samβada landscape genomic tool implemented into the R package R.SamBada v. 0.1.3 (Duruz et al. 2019) by computing multivariate models between a binary genetic variable and multiple environmental variables and assessing the significance against a null model (Stucki et al. 2017). Biallelic SNPs may have the maximum three distinct genotypes (e.g., *A/A*, *A/T*, and *T/T*). Genotype data were pruned using the function *prepareGeno* integrated into R.SamBada's pipeline, which relies on the snprelate package v. 1.32.0 (Zheng et al. 2012). SNPs with missing data (MD) > 20% and minor allele frequency (MAF) < 5% were excluded from the analysis, reducing the genotype input data from 124,262 SNPs to 8604 SNPs, comprising 25,812 genotypes (Figure S3b,c). Population structure was assessed by means of a principal component analysis (PCA) implemented in snprelate integrated into the *prepareEnv* function of R.SamBada.

**FIGURE 2:**
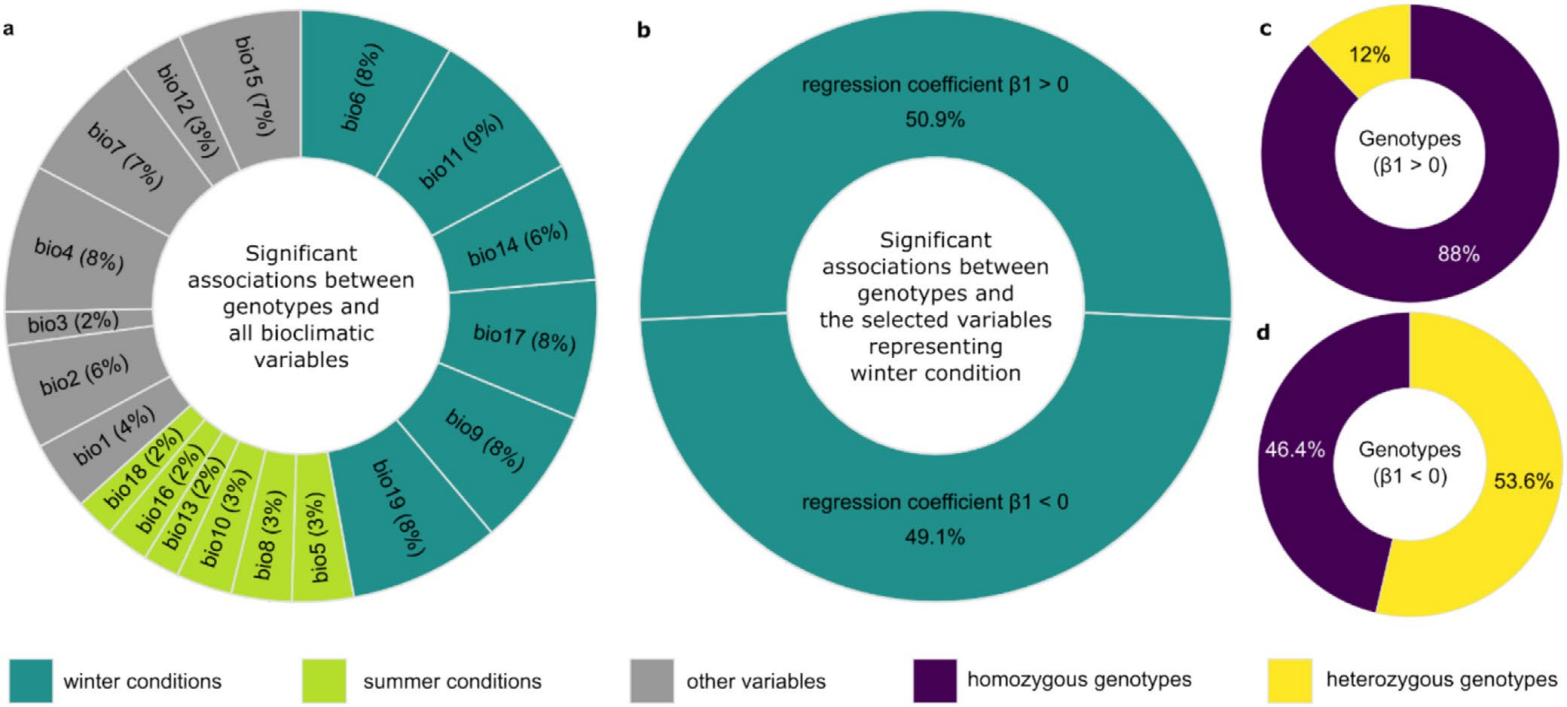
Genotype‐environment associations (GEAs) results. (a) Percentage of significant associations (*q*‐valueG < 0.05 and *p*‐valueW < 0.05) between single nucleotide polymorphism (SNP) variation and 19 WorldClim bioclimatic variables. (b) Number of genotypes significantly associated with the selected variables representing winter conditions (Bio6, Bio11, Bio14, and Bio17) with positive or negative regression coefficient *β*1. Percentage of homozygous and heterozygous genotypes (highlighted by violet and yellow colors, respectively) among the positively (c) and negatively (d) associated genotypes from chart (b).

The first run was made with all 19 bioclimatic variables with no correlation threshold to screen the number of associations with each variable (Figure 2a, Table S4). A second run was conducted using four highly correlated variables (Bio6, Bio11, Bio14, Bio17) (Figure S2), including two representing the temperature of the coldest period (Bio6: Min Temperature of Coldest Month, Bio11: Mean Temperature of Coldest Quarter) and two representing the precipitation of the driest period (Bio14: Precipitation of Driest Month, Bio17: Precipitation of Driest Quarter) (Figure 2b, Tables S5 and S6). Although spring represents the driest period (Figure S1), we adopted the terminology “cold and dry winter conditions” to conveniently describe the extremes of low temperature and precipitation. These variables are closely clustered (Figure S2), making them inseparable, and their temporal boundaries may also be poorly defined. Statistical significance of GEAs was assessed by the function *prepareOutput* integrated into R.SamBada, providing *p*‐ and *q*‐values (Duruz et al. 2019). Associations with both G‐score‐based *q*‐values < 0.05 and Wald‐score‐based *p*‐values < 0.05 were considered significant. The median allele frequency was calculated for each of the significantly associated genotypes (Table S7). The percentage of homozygosity and heterozygosity was separately calculated for both groups of significantly associated genotypes with positive (*β*1 > 0) and negative (*β*1 < 0) regression coefficient (Table S7).

Spatial autocorrelation integrated into R.SamBada was measured using Moran's *I* for genotypes and environmental variables with a weighting scheme of 20 neighbors. Pseudo *p*‐values for the permutations‐based significance test of global and local measures of spatial autocorrelation were computed with 999 permutations (Data S1).

### 2.5 Alignment and Annotation

The nucleotide sequences harboring significantly associated SNPs were extracted from the ipyrad output (Table S8) and aligned to the *L. sibirica* nuclear reference genome (Bondar et al. 2022; Kuzmin et al. 2019) (NCBI GenBank accession number GCA_004151065.1) using Bowtie 2 v. 2.3 in the “–local” mapping mode with default parameters (Langmead and Salzberg 2012). The overall alignment rate was 100%. The *L. sibirica* nuclear genome annotation file (gff3) was used to annotate the alignment file (bam) using BEDTools intersect (Table S9) (Quinlan and Hall 2010). SNPs in the mapped loci annotated as gene regions are listed in Table S10; however, only the ones with available gene names were considered for further detailed analysis.

### 2.6 GO Enrichment Analysis

Overrepresentation of GO categories was assessed by a hypergeometric test of the number of annotated candidate adaptive genes with significantly associated SNPs (cluster frequency) and all 2407 annotated genes (approximately 6% of the genes found in a larch nuclear genome, [Bondar et al. 2022; Kuzmin et al. 2019]) retrieved by annotating all GBS‐assembled loci (total frequency) (Table S11), using the Cytoscape plugin BiNGO (Maere, Heymans, and Kuiper 2005; Shannon et al. 2003). The “whole annotation as reference set” and the “GO_Full” ontology file were used in BiNGO. The full results of GO enrichment analysis reported in Table S12 represent biological process, molecular function, and cellular component GO terms. To account for multiple SNPs in the same gene, a progressing number was added after the gene name for each additional SNP (e.g., gene_1, gene_2). Therefore, each gene name represents a single SNP, and the resulting enrichment *p*‐values might be slightly higher due to the higher total frequency in the custom annotation gene list. Due to deleted obsolete GO categories, 16 and 6 genes were excluded from the analysis among the candidate adaptive and the custom reference annotation gene lists, respectively. GO categories with a *p*‐value < 0.05 were considered significant. False discovery rate (FDR) corrected *p*‐values (Benjamini and Hochberg 1995) are presented in Table S12. For SNPs in the same gene, the enrichment analysis was tested considering only one SNP per gene (Table S13).

### 2.7 Reduction and Visualization of GO Terms According to Semantic Similarity

The significantly enriched GO terms (*p* < 0.05) belonging to the biological process sub‐ontology were reduced by removing redundant GO terms and clustered by the SimRel semantic similarity measure using REVIGO (Supek et al. 2011). The whole Uniprot database was used, and obsolete GO terms were removed. The choice of the groups' representatives was guided by the *p*‐values obtained from the GO enrichment analysis. The treemap produced by REVIGO is reported in Figure S4a. The clustering results were further summarized according to the most shared cluster representative GO terms by the genes included in all clusters (Table S14) and represented in the treemap of Figure 4 created in R using the package treemap v. 2.4.4. The treemap produced by REVIGO, considering only one SNP per gene, is reported in Figure S4b. The graphic reconstruction presented in Figure 5 was created using pictograms designed by Macrovector and Barudakvisual available from the Freepik online platform.

## 3 Results

### 3.1 Genotyping, Population Structure and GEA Analysis

From genotyping 243 individual *Larix* trees (196 *L. cajanderi* and 47 *L. gmelinii* ) (Figure 1a, Table S1), we obtained a median of 1,472,510 raw reads assembled into 15,201 loci per sample and a total of 124,262 SNPs with an average of 1.3% of estimated heterozygous sites per individual tree (Table S3).

The cross‐validation procedure showed that the number of clusters (*K*) with the lowest error was *K* = 2, which is in agreement with having two larch species and their potential hybrids in our samples (Figure S5). The proportion of ancestry resulting from the admixture analysis revealed a genetic structure in accordance with the west–east longitudinal gradient (Figure S6). Interestingly, the hybrid individuals classified as *L. cajanderi* inhabiting the large species boundary area of central Yakutia were genetically closer to the western *L. gmelinii* individuals rather than *L. cajanderi* from the far east Chukotka (Figure 1a).

GEAs were assessed by logistic regressions between the filtered input data of 25,812 genotypes (details in Materials and Methods) and the bioclimatic variables at the sampling locations obtained from the WorldClim data (Table S2) (Duruz et al. 2019; Fick and Hijmans 2017; Stucki et al. 2017). To mitigate the risk of spatial dependency‐induced false positives, population structure was incorporated into the GEA analysis by PCA. The substantial variance difference between the first (~0.075) and subsequent PCA components (~0.019, ~0.012) suggested that the first component primarily represented population structure (Figure S3a). Consequently, the first component's scores were incorporated into the GEA multivariate analysis (plotted in Figure 1c), resulting in 4018 genotypes significantly associated with at least one of the 19 bioclimatic variables, yielding a total of 16,434 significant GEAs (*q*‐valueG < 0.05; *p*‐valueW < 0.05) (Table S4).

### 3.2 Cold and Dry Winter Conditions as Adaptation Drivers

According to the WorldClim data recorded at the individual sampling location (Fick and Hijmans 2017), winter and spring exhibited the lowest temperatures and precipitation values, considered in this study to represent the coldest and driest periods. In contrast, summer showed the highest temperatures and precipitation, indicating the warmest and wettest period (Figure S1). Our results showed 7744 (47.1%) genotypes significantly associated (*q*‐valueG < 0.05; *p*‐valueW < 0.05) with at least one of the six environmental variables representing cold and dry winter conditions, and 2661 (16.2%) genotypes associated with the six variables representing warm and wet summer conditions (Figure 2a, Tables S4 and S5).

Due to the high correlation of bioclimatic variables, as demonstrated by their close clustering (Figure S2), it was not possible to separate the influence of low temperature and low precipitation. Thus, we selected four highly correlated variables (*r* > 0.6) as the most representative to study the effect of the coldest and driest conditions on the genotype (Bio6, Bio11, Bio14, and Bio17) (Figure 2b, Table S5).

**FIGURE 3:**
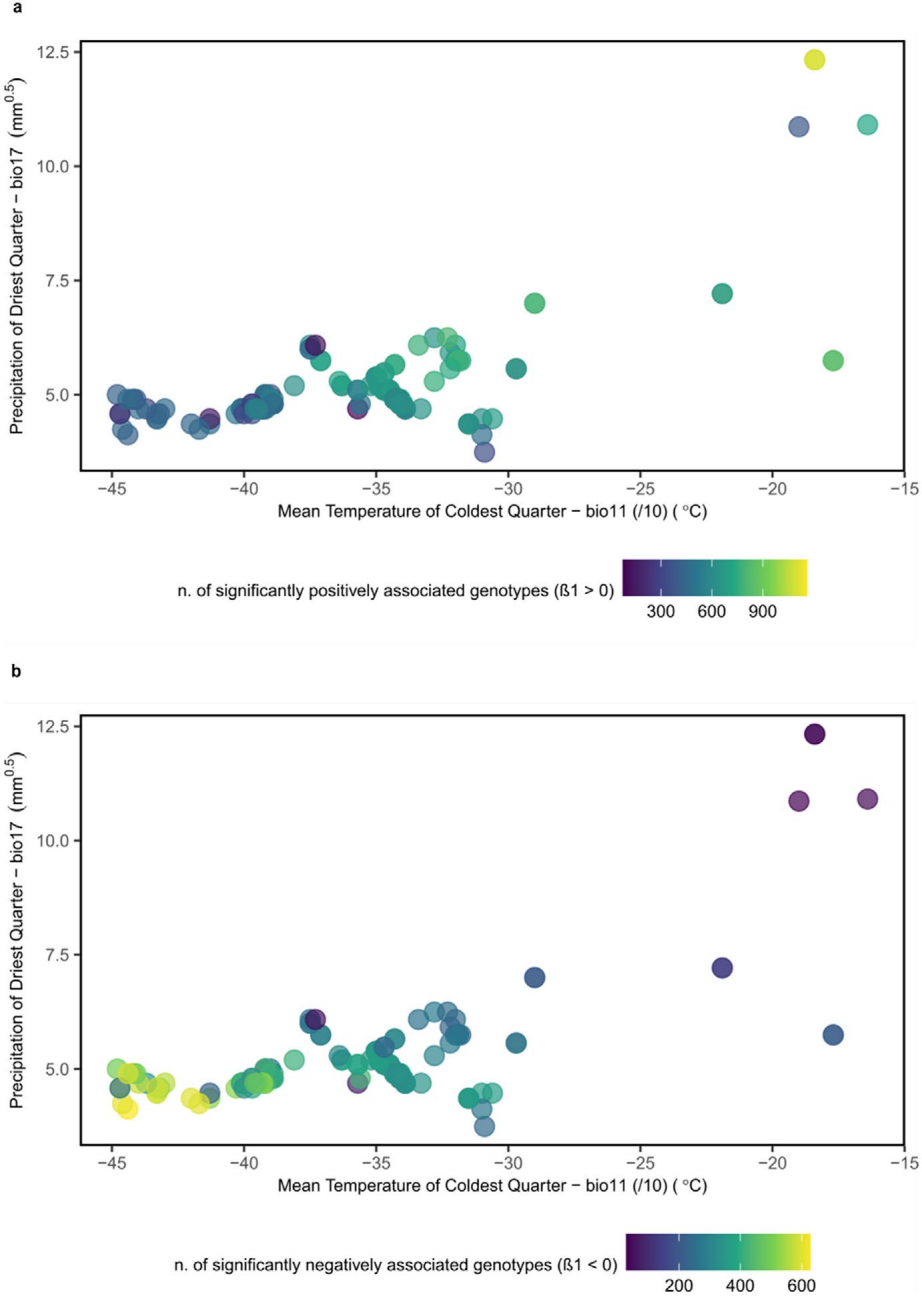
Dots represent individuals plotted according to the values of precipitation of driest quarter (Bio17) and mean temperature of coldest quarter (Bio11) at the sampling location. Color represents the number of significantly associated genotypes with the group of four selected variables representing winter conditions with (a) a positive regression coefficient (*β*1 > 0) and (b) a negative regression coefficient (*β*1 < 0).

We obtained a total of 2622 genotypes significantly associated (*q*‐valueG < 0.05; *p*‐valueW < 0.05) (Table S6) with at least one of the selected bioclimatic variables. Among these, 1334 genotypes (50.9%) with a positive regression coefficient (*β*1 > 0) are mostly homozygous (88%) for major alleles with a median allele frequency of 52.7% (Figures 2c and 3a, Table S7), while 1288 genotypes (49.1%) with a negative regression coefficient (*β*1 < 0) – indicating their enrichment in individual trees located in the coldest and driest regions (Figure 3b)—are mostly heterozygous (53.6%) with a median minor allele frequency of 17.5% (Figure 2d, Table S7). The distribution of the genotypes of each individual on the geographical map is represented in Figure S7.

### 3.3 Biological Processes Underlying Genetic Adaptation to Winter Cold and Dry Winter Conditions

**FIGURE 4:**
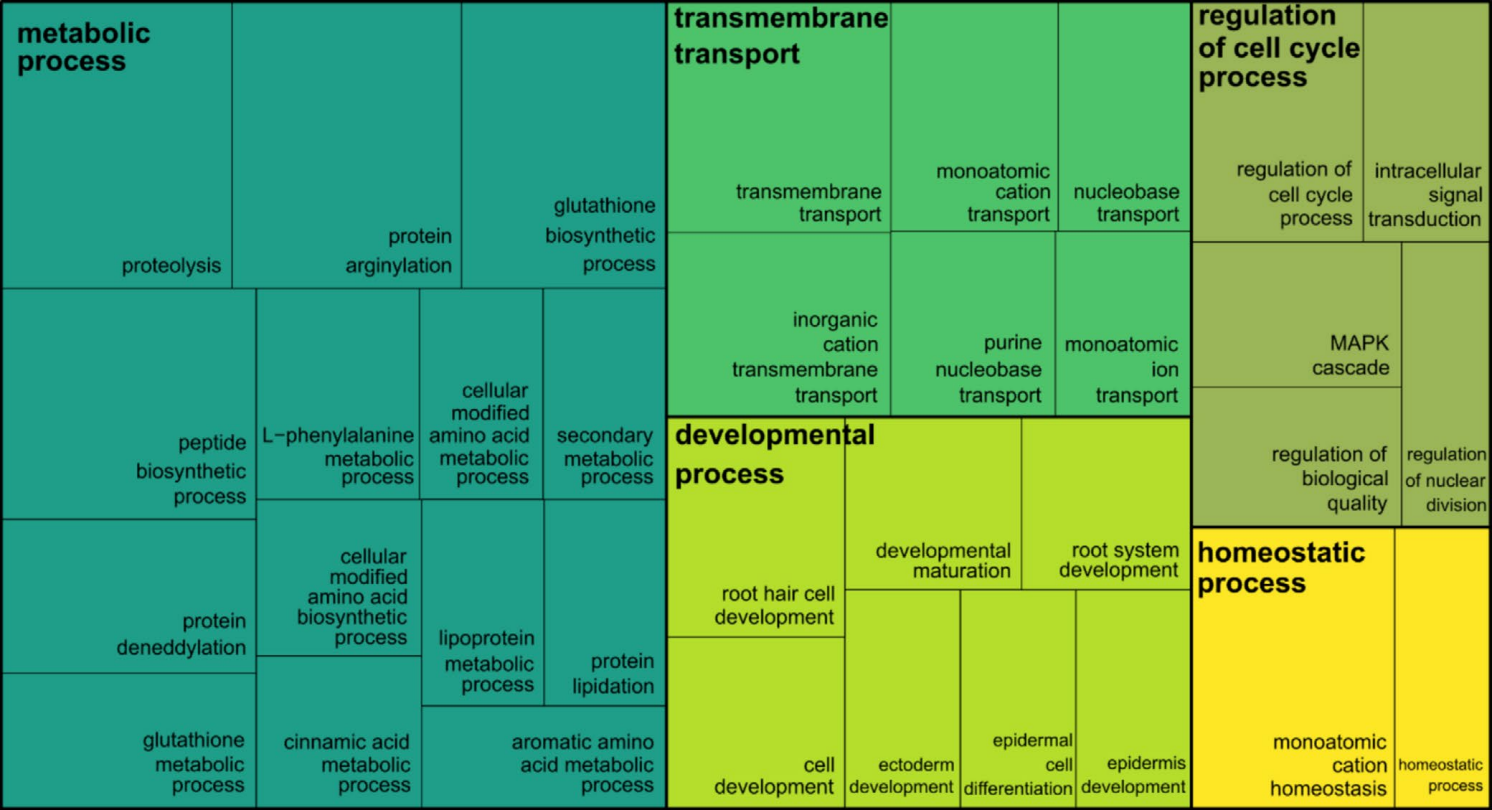
Reduction and visualization of gene ontology (GO) terms according to semantic similarity. Treemap summarizing biological processes enriched by candidate adaptive genes associated with strict winter conditions. Each rectangle represents a significantly (*p* < 0.05) over‐represented GO term. The size of each rectangle is proportional to the GO enrichment analysis based on Log10(*p*‐value) for that category. The five colors represent the five summarized GO term clusters, respectively.

The loci harboring the genotypes associated with strict winter conditions (Table S8) were aligned and annotated using the published *L. sibirica* nuclear reference genome and its annotation (Table S9) (Bondar et al. 2022; Kuzmin et al. 2019). This resulted in 259 SNPs (232 in exons, 17 in introns, and 10 in 5′‐ and 3′‐untranslated regions (UTRs)) across 184 genes with available gene name and GO ID annotations (Table S10), on which GO enrichment analysis was performed (Table S11), resulting in 134 significant GO terms (*p* < 0.05) (Table S12). The GO terms belonging to the biological process sub‐ontology were clustered by semantic similarity measures and reduced to 11 representative GO terms (Figure S4a). The clustering results were further summarized into five main biological processes: (1) metabolic process, (2) transmembrane transport, (3) developmental process, (4) regulation of cell cycle process, and (5) homeostatic process (Figure 4, Table 1; full data are presented in Table S14).

**TABLE 1:** Candidate adaptive genes grouped according to the significantly enriched gene ontology (GO) terms.

Cluster representative GO term	Gene name	Description	Gene region (number of SNPs)
Metabolic process	E5FQ11	Arogenate dehydratase	Exon (1)
	A0A1I9WKA5	Arogenate dehydratase	Exon (1)
	PAL1	Phenylalanine ammonia‐lyase	Exon (1)
	B8LR86	Phenylalanine ammonia‐lyase	Exon (1)
	C4H1	Trans‐cinnamate 4‐monooxygenase‐like	Exon (1)
	C2845_PM18G11900	Glutathione synthetase, chloroplastic isoform X1	Exon (1), Intron (2)
	GSTL3	Lambda class glutathione S‐transferase	Exon (1)
	D5086_0000111890	Arginyl‐tRNA—protein transferase 2	Exon (4)
	VITISV_029713	Multicopper oxidase	Exon (1)
	A0A291FB12	Putative hydroxylase	Exon (5)
	B296_00014275	Geranylgeranyl transferase type‐2 subunit alpha 1 isoform X1	Exon (1)
	TORRG33X02_197710	Glycylpeptide N‐tetradecanoyltransferase 1‐like	Exon (1)
Transmembrane transport	COLO4_21754	Cation/H(+) antiporter 19	Exon (1)
	LOC109005674	Cation/H(+) antiporter 19‐like	Exon (3)
	AQP	Probable aquaporin	Intron (2)
	A9NZ54	Aquaporin TIP1.1	Exon (1)
	A9NNZ4	Aquaporin PIP1‐2	Exon (1)
	C2845_PM15G21170	Calcium‐transporting ATPase 10, plasma membrane‐type isoform X1	Intron (2)
	VIT_04S0023G01860	Sugar transporter ERD6‐like 6	Exon (1)
	Q1XG57	Putative phosphate transporter	Exon (2)
	C5167_002500	Cationic amino acid transporter 7, chloroplastic‐like	Exon (1)
	RCHIOBHM_CHR1G0371841	Adenine/guanine permease AZG1	Exon (2)
	LOC104600132	Spinster protein	Exon (1)
	LOC106774067	Organic cation/carnitine transporter 1 isoform X2	Exon (1)
	TRITD_6BV1G096050	Unnamed protein product	Intron (1)
	BVC80_695G35	Choline transporter‐like	Exon (2)
	COLO4_12854	ABC transporter G family member 40	Intron (1)
	C3L33_11864	Metal tolerance protein 1 isoform X2	Exon (1)
Developmental process	B8LL91	Arabinosyltransferase RRA3‐like	Exon (2)
	ES288_A07G059700v1	Non‐specific phospholipase C6‐like	5′ (1), Exon (1)
	C3HDZ2	Homeobox‐leucine zipper protein ATHB‐15‐like	Exon (1)
Regulation of cell cycle process	JCGZ_04406	COP9 signalosome complex subunit 4	5′ (2)
	AXG93_412S1310	Mitotic checkpoint serine/threonine‐protein kinase BUB1	Exon (2)
Homeostatic process	COLO4_21754	Cation/H(+) antiporter 19	Exon (1)
	LOC109005674	Cation/H(+) antiporter 19‐like	Exon (3)
	B8LRJ7	Hexokinase	Exon (1)
	PIF1	ATP‐dependent DNA helicase PIF1‐like	Exon (1)

*Note:* For each gene, the representative GO term, the description, the single nucleotide polymorphism (SNP) region, and the number of candidate adaptive SNPs in the respective gene are reported.

## 4 Discussion

### 4.1 Population Structure

The debate on classifying *L*. *cajanderi* as either a distinct species or a subspecies of *L. gmelinii* is still ongoing, considering their likely divergence from a common ancestor during the late Pleistocene (Abaimov 2010). Admixture analysis reveals that hybrid populations reside within the large boundary area of central Yakutia, the coldest region. This evidence suggests that hybrids might have enhanced survival capabilities in harsh environments, potentially through adaptive introgression. Hybrid individuals classified as *L*. *cajanderi* are genetically more closely related to *L. gmelinii* from the west than to *L*. *cajanderi* from the far east Chukotka, a pattern likely influenced by geographical barriers. This finding highlights the necessity of analyzing both *Larix* species together to study their genetic adaptation to environmental conditions. While combining species and their hybrids could inflate the number of significant SNPs demonstrating GEAs, potentially introducing false positives due to selectively neutral population structure, we addressed this issue by incorporating covariates representing population structure from PCA analysis into the Samβada model.

### 4.2 Cold and Dry Winter Conditions as Adaptation Drivers

Our GEA results show that the majority of candidate adaptive SNPs are associated with the bioclimatic variables reflecting winter conditions rather than summer conditions. These results suggest that winter cold and dry winter conditions are likely the main drivers of genetic adaptation of *Larix* in eastern Siberia.

Eastern Siberian larches are well adapted to cold and dry winter conditions; indeed, their deciduousness and shallow roots indicate that winter desiccation and frozen soils strongly impact their adaptation to avoid severe water deficit (Abaimov 2010; Miyahara et al. 2011; Neale and Wheeler 2019, 171). However, recent physiological adaptation studies, promoted by potential effects of global warming, suggest that larch tree growth is limited by soil‐water deficit due to an ongoing reduction of summer precipitation and permafrost degradation (Sidorova et al. 2021; Miyahara et al. 2011; Ohta et al. 2008; Peng et al. 2022; Tchebakova et al. 2023). Nevertheless, our results suggest that extreme winter conditions remain the primary environmental stressor among the selective factors driving the adaptation of *Larix*. The observed genetic variation associated with WorldClim bioclimatic variables recorded between 1970 and 2000 reflects both short‐term and long‐term selection acting on the current and recent generations of larch in the genotyped trees. Furthermore, prior evidence indicates a general cooling trend from the Late Holocene (~4500 BP) until the nineteenth century, particularly in the Northern Hemisphere north of 60° N (Bader et al. 2020; Kaufman and Broadman 2023). This likely suggests a comparable trend of both short‐term and long‐term climate as selecting factors driving larch adaptation over time.

We found that the genotypes correlated with strict winter conditions are mainly carried by the hybrid individuals located in the species boundary zone of central Yakutia. In these harsh environments, selection appears to favor minor alleles, enhancing heterozygosity—a prerequisite for evolutionary adaptation as indicated by previous research (Chung et al. 2023). Therefore, hybrid trees located in regions with extreme cold and dry winter conditions, such as central Yakutia, are likely undergoing significant genetic adaptation to these stresses.

### 4.3 Biological Processes Underlying Genetic Adaptation to Winter Cold and Dry Winter Conditions

By identifying candidate adaptive genes and conducting GO enrichment analysis, we found biological processes that may underlie the genetic bases of important adaptive traits for larch growth and survival in response to cold and dry winter conditions. Earlier research suggests that conifer populations from regions with cold winters exhibit significant tolerance to both cold and drought stresses (Neale and Wheeler 2019, 171). Additional findings showed shared physiological impacts of freezing and drought in conifers, including disruption of hydraulic functions (McCulloh et al. 2023). Our results support these observations, as the candidate adaptive genes exhibit potential overlapping roles in responding to both stressors, indicating potential shared mechanisms of adaptation.

Since the individual trees included in our study belong to different species ( *L. gmelinii* and *L. cajanderi*) that are spread across a large geographical range, we might assume that both parallel evolution and local adaptation are operative in their genomes. The candidate adaptive SNPs in genes involved in the five enriched biological processes might thus indicate a polygenic parallel evolution of important adaptive traits in response to dry, cold winter conditions across the two *Larix* species (Barghi, Hermisson, and Schlötterer 2020; McCulloh et al. 2023). However, further studies using genome‐wide approaches will be necessary to study polygenicity in functional categories.

#### 4.3.1 Metabolic Process

Our study identified an enrichment by adaptive SNPs in 12 candidate genes involved in metabolic processes (Table 1). Four genes play a critical role in the biosynthesis of phenylalanine (Phe), in particular, the genes *E5FQ11* and *A0A1I9WKA5*, encoding an arogenate dehydratase (ADT), and the genes *PAL1* and *B8LR86*, encoding a phenylalanine ammonia‐lyase (PAL). ADT catalyzes the conversion of arogenate into phenylalanine, that is converted by PAL enzymes into cinnamic acid, which is the first component of the phenylpropanoid pathway, generating precursors essential for the synthesis of lignin, phenylpropanoids, and flavonoids. These compounds are vital for plant reproduction, growth, development, and defense against both biotic and abiotic stressors (Bondar et al. 2022; Pascual et al. 2016). The gene *C4H* with adaptive associations, potentially encoding for a trans‐cinnamate 4‐monooxygenase, also plays a role in lignin biosynthesis in roots under stress conditions (Li et al. 2022). The thickening of the secondary cell wall through lignin deposition was found to improve resistance to cold, drought, and salt stress by reducing water permeability and maintaining cell turgor under water‐limited conditions in several plants (Bagal et al. 2012; Dong and Lin 2021).

Furthermore, we found four adaptive SNPs in two genes involved in the glutathione metabolic process, specifically, the genes *C2845_PM18G11900* and *GSTL3*, encoding for chloroplastic glutathione synthase and lambda class glutathione S‐transferase, respectively. Biotic and abiotic stresses increase reactive oxygen species (ROS) production, against which glutathione (GSH), the most prevalent plant cell antioxidant, plays a critical role by scavenging ROS with its active sulfhydryl group (Dorion, Ouellet, and Rivoal 2021). The studies of *L. kaempferi* and other plants indicated that environmental stresses influence the expression of the GSH synthase and the GST genes, potentially supporting normal growth and development (Dorion, Ouellet, and Rivoal 2021; Hasanuzzaman et al. 2017; Li, Lee, et al. 2021; Zhang et al. 2021). Therefore, our evidence suggests that metabolic processes such as the phenylalanine and glutathione biosynthesis might have significant roles in enhancing *Larix* tolerance to the extreme cold and dry winter conditions in eastern Siberia.

#### 4.3.2 Transmembrane Transport and Homeostatic Processes

Our findings revealed an enrichment by adaptive SNPs in 16 candidate genes involved in transmembrane transport processes and four genes involved in homeostatic processes (Table 1). These genes exhibit convergent roles in maintaining the osmotic balance of water and ions, a crucial mechanism for stress tolerance, especially under cold and dry conditions. The genes *COLO4_21754* and *LOC109005674*, encoding a cation/H^(+)^ antiporter 19, are involved in the homeostasis of K^+^ (Ali et al. 2020; Jia et al. 2018; Li, Luo, et al. 2021). The K^+^ deficiency has been observed under drought, salinity, and frost stresses due to the decrease of K^+^ diffusion in the soil towards the roots (Wang et al. 2013). The genes *AQP*, *A9NZ54*, and *A9NNZ4* encode aquaporins, particularly *TIP1.1* and *PIP1‐2*, involved in membrane water permeability control, important in cold hardiness and freeze‐induced dehydration stress resistance (Baldi and La Porta 2022; Gill et al. 2021; Neale and Wheeler 2019, 111; Rahman et al. 2020). The gene *C2845_PM15G21170*, encoding the calcium‐transporting ATPase 10, is involved in Ca^2+^ homeostasis, particularly in maintaining the differential Ca^2+^ concentrations in cellular compartments (Yadav 2021). Plants possess a rapid stress signaling system based on Ca^2+^ waves that propagate through the plant and participate in nearly all aspects of plant growth and development (Choi et al. 2014; Ishka et al. 2021). The gene *B8LRJ7* encodes a hexokinase involved in intracellular glucose homeostasis (Pérez‐Díaz et al. 2021). The gene *VIT_04S0023G01860*, potentially encoding the sugar transporter ERD6, is considered to take part in the redistribution of sugars to protect plant cells from the impacts of dehydration and cold stress (Slawinski et al. 2021).

There is evidence that drought stress increases salt and ion concentrations in the larch forest soils of eastern Siberia (Basu et al. 2016; Herzschuh et al. 2013). This is likely to cause a decreased ion diffusion in the soil towards the roots, inducing hyperosmotic stress (Li, Luo, et al. 2021). Therefore, the maintenance of the osmotic balance of water and ions to provide a correct inflow of nutrients and a functional signaling network might be crucial for the adaptation of larches in eastern Siberia.

#### 4.3.3 Developmental Process

Our findings highlight an enrichment by adaptive SNPs in three candidate adaptive genes with putative roles in root development and stress‐response mechanisms (Table 1). The *ES288_A07G059700V1* gene, encoding the nonspecific phospholipase C6 (NPC6), plays a potential role in gametophyte development, root growth, and drought response mechanisms triggered by cellular osmolarity changes due to water loss (Ali et al. 2022; Beck et al. 2007; Nakamura and Ngo 2020; Ngo, Kanehara, and Nakamura 2019). The gene *B8LL91*, encoding for the arabinosyltransferase RRA3‐l, is involved in the formation of extensins (EXTs) in root hair cell walls, crucial for nutrient uptake (Velasquez et al. 2011). The gene *C3HDZ2*, encoding a homeobox‐leucine zipper protein ATHB‐15‐like transcription factor, is implicated in regulating vascular development (Du et al. 2015; Kim et al. 2005).

Although the shallow roots of central and eastern Siberian larches are well adapted to continuous permafrost (Abaimov 2010), previous studies have found that frozen soils limit moisture uptake by the roots and repeated freeze–thaw cycles in the soil active layer affect the root system development (Miyahara et al. 2011). Here, we found genetic evidence that the adaptation of root developmental processes could be essential for larch trees to cope with frozen soils and associated potential drought conditions in eastern Siberia.

#### 4.3.4 Candidate Adaptive SNPs in Untranslated Gene Regions

Increasing evidences show the importance of 5′‐ and 3′‐UTRs in post‐transcriptional regulation of mRNA properties such as stability, transport, and translation efficiency, which are critical in the activation of stress tolerance and developmental mechanisms (Srivastava et al. 2018). We have found seven candidate adaptive SNPs located in the 5′‐UTR region and one candidate adaptive SNP located in the 3′‐UTR of different genes. Among them, the gene *ACMD2_22301*, encoding an Actin‐7‐like protein, is involved in the regulation of primary root elongation and thermoresponse in *Arabidopsis* (Parveen and Rahman 2021). Adaptive candidate genes with available annotation are reported in Table S14.

## 5 Conclusions

### 5.1 Implications for Future *Larix* Adaptation

Our data show that cold and dry winter conditions may be the greatest challenge for *Larix* in eastern Siberia. From this perspective, global warming might cause more favorable conditions for growth in the coldest areas of Yakutia as well as an expansion of *L. gmelinii* and *L. cajanderi* species ranges to the northeast. However, recent studies seem to indicate an ongoing increase of continental climate conditions in eastern Siberia with increasing extremes of winter cold and summer warmth and an overall decrease of precipitation (Sidorova et al. 2021; Miyahara et al. 2011; Ohta et al. 2008; Tchebakova et al. 2023; Wang et al. 2022). In this scenario, the larches of central Yakutia might undergo a stronger challenge due to cold, drought, and accumulation of salt with possible contraction or density reduction. The rate at which these changes might occur will determine the strength of their impact on the larches' adaptation (Gloy, Herzschuh, and Kruse 2023). To understand the adaptive potential of larch to adjust to global warming or an increasing continental climate, further research should be conducted on the current selective factors in different ecological niches as well as to identify the potential of polygenic local and parallel adaptation responses. A high‐resolution, genome‐wide approach on a spatially balanced sampling plan will therefore be necessary.

**FIGURE 5:**
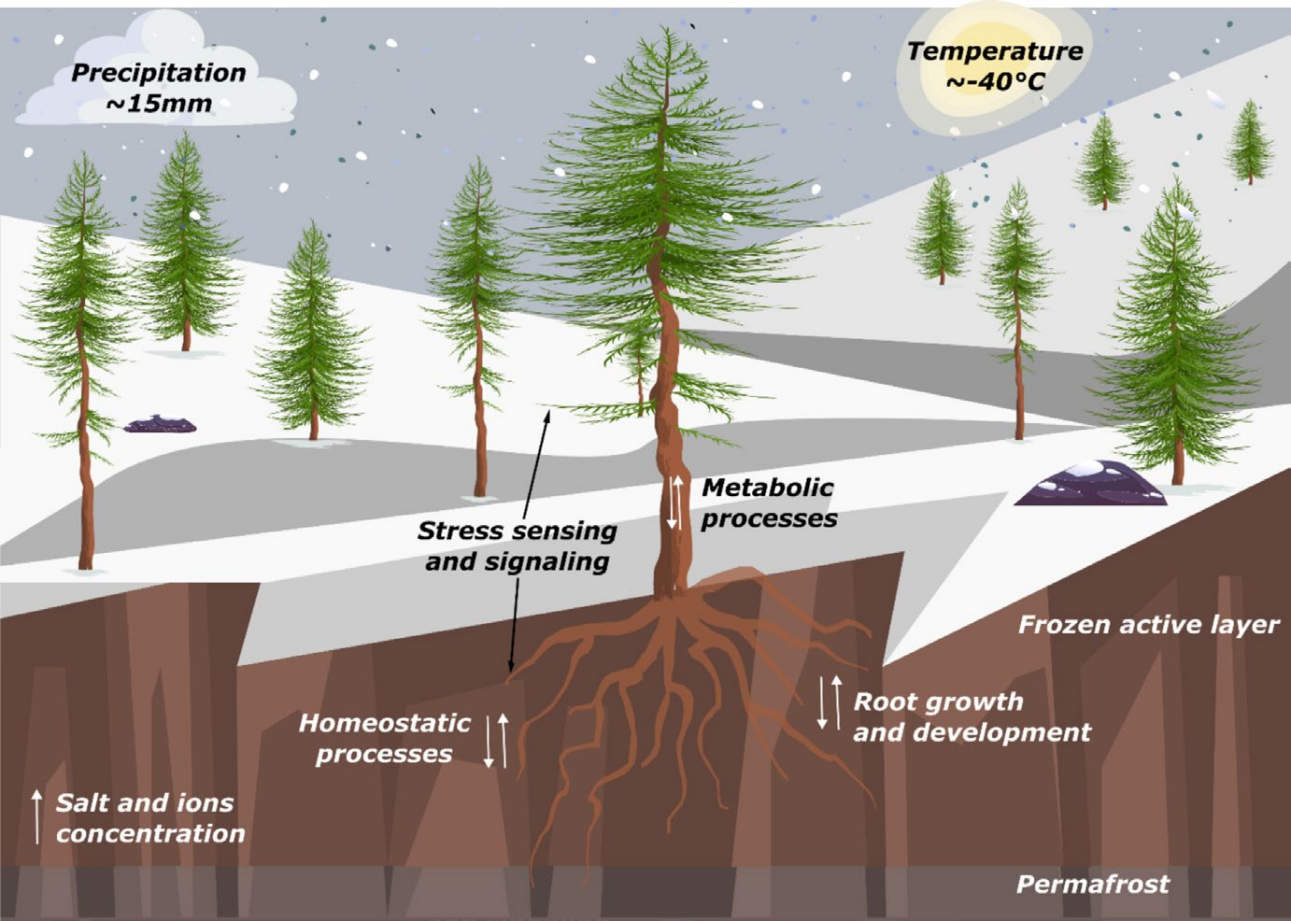
Graphic reconstruction of the eastern Siberian winter environment and *Larix* adaptation mechanisms to cold and drought identified in our study.

In conclusion, our study indicates that the continental climate conditions exert selective pressure on larch, particularly in hybrid individuals found within the species boundary zone in central Yakutia. These individuals may have evolved enhanced stress resistance and tolerance to the extremely harsh conditions of these regions, possibly through adaptive introgression. The region's prolonged and severe winter, with frozen soils and potential freeze‐induced drought, has challenged metabolic processes, such as the biosynthesis of phenylalanine and glutathione, the osmotic balance, the transport of ions and nutrients, as well as the growth and development of the larch root system (Figure 5). The candidate adaptive SNPs identified in our study could be further investigated to determine whether they represent nonsynonymous substitutions, with the potential to explore signatures of positive or negative selection using neutrality tests. Additionally, these SNPs could serve as a valuable resource for developing genotyping arrays to screen for adaptive traits. Upon further validation, our findings may also contribute to selective breeding efforts aimed at enhancing resilience in cultivation or reforestation programs.

## Author Contributions

**Stefano Meucci:** conceptualization (lead), data curation (equal), formal analysis (lead), investigation (lead), methodology (lead), project administration (equal), software (equal), validation (equal), visualization (lead), writing – original draft (lead), writing – review and editing (equal). **Stefan Kruse:** conceptualization (equal), formal analysis (equal), investigation (equal), methodology (equal), project administration (supporting), resources (equal), software (equal), validation (equal), writing – review and editing (equal). **Sarah Haupt:** investigation (equal), resources (equal). **Kathleen R. Stoof‐Leichsenring:** project administration (supporting), resources (equal). **Konstantin V. Krutovsky:** formal analysis (supporting), methodology (supporting), writing – original draft (supporting), writing – review and editing (supporting). **Nadine Bernhardt:** data curation (supporting), methodology (supporting). **Dörte Harpke:** formal analysis (supporting), investigation (equal), resources (equal). **Ulrike Herzschuh:** conceptualization (equal), formal analysis (equal), funding acquisition (lead), methodology (equal), project administration (lead), resources (equal), supervision (lead), writing – original draft (equal), writing – review and editing (equal).

## Conflicts of Interest

The authors declare no conflicts of interest.

## Supporting information


**Figure S1** Distribution of historical climate data. Seasonal (a) and annual (b) distribution of the median precipitation (mm), and minimum and maximum temperature (°C) measured at the sampling locations.
**Figure S2** Correlation analysis and hierarchical clustering among bioclimatic variables (Bio1–Bio19) and geographical coordinates. Only significant correlations (*p* < 0.01) are represented. Descriptions of the bioclimatic variables are in Table S5.
**Figure S3** Genotype pruning histograms and population structure assessed by PCA in R.SamBada. (a) Histogram of missingness with the vertical red line representing the threshold value of 0.2. (b) Histogram of minor allele frequency with the vertical red line representing the threshold value of 0.05. (**c**) Variance proportion of the first 100 PCA axes representing population structure with the vertical red line indicating the first PCA component included in R.SamBada multivariate analysis.
**Figure S4** Reduction and visualization of GO Terms according to semantic similarity. Treemap produced by REVIGO for biological processes considering multiple SNPs per gene (a) and one SNP per gene (b). Each rectangle represents a significantly (*p* < 0.05) over‐represented GO term. The size of each rectangle is proportional to the GO enrichment analysis based on Log10(*p*‐value) for that category. The four colors represent the four summarized GO term clusters represented in Figure 4, respectively; GO terms represented in white were not included in the summary. Detailed results are presented in Table S14.
**Figure S5** Cross‐validation procedure. Plot displaying the results of ADMIXTURE cross‐validation error considering 5 possible clusters.
**Figure S6** Admixture analysis. The proportion of admixtures based on two clusters (*K* = 2) were plotted along the longitude values of each individual location. The IDs of the individual tree were colored according to the assigned species taxonomy (*L*. *gmelinii* in blue, *L*. *cajanderi* in black).
**Figure S7** Genotypes’ distribution. Map of the study area with background black‐white shade representing the WorldClim bioclimatic variable Mean Temperature of Coldest Quarter (Bio11). Dots represent the individual sampling sites. Color dots represent the number of significantly associated genotypes with the group of four selected variables representing winter condition with (a) positive regression coefficient (*β*1 > 0), and (b) negative regression coefficient (*β*1 < 0). Individuals were spread on the map to allow visibility of all samples with color reflecting the number of genotypes.
**Figure S8** Scatterplots visualizing the relationships between heterozygosity (H), deviation (D), and allele ratio before (using Stacks maximum mismatch threshold of 3) and after applying HDplot filtering thresholds. Panel (a) shows the distribution of loci based on H and D prior to filtering, with dashed red lines indicating the thresholds for deviation (|D| ≤ 7). Panel (b) presents the retained loci after filtering. Panels (c) and (d) illustrate the relationship between H and allele ratio before and after filtering, respectively.

**Table S1** Overview of sampled individual trees and geographic coordinates.
**Table S2** WorldClim bioclimatic variables (Bio1–Bio19) and historical climate data (prec, minT, maxT) recorded at the sampling locations. Variance proportion of the first PCA component to account for population structure (pop).
**Table S3** Summary results of loci assembled with ipyrad (for a detailed explanation of each data column, refer to the ipyrad documentation).
**Table S4** Full results of significant (*q*‐valueG < 0.05 and *p*‐valueW < 0.05) GEAs considering all bioclimatic variables.
**Table S5** Overview of GEAs results for each bioclimatic variable.
**Table S6** Full results of significant (*q*‐valueG < 0.05 and *p*‐valueW < 0.05) GEAs considering four selected bioclimatic variables (Bio6, Bio11, Bio14, Bio17) representing winter conditions.
**Table S7** Allele frequencies of genotypes associated with positive (*β*1 > 0) or negative (*β*1 < 0) regression coefficients.
**Table S8** Loci sequences (listed according to the loci number) containing the candidate adaptive SNPs associated with strict winter conditions (*β*1 < 0).
**Table S9** Full annotation results obtained by intersecting the mapping file (bam) and the annotation file (gff3) from the *Larix sibirica* nuclear genome reference (NCBI GenBank accession number GCA_004151065.1).
**Table S10** Overview of SNPs and respective genotypes significantly associated with strict winter conditions (*β*1 < 0) located within genes.
**Table S11** Candidate adaptive gene and custom annotation gene lists used for GO enrichment analysis.
**Table S12** Full GO enrichment analysis results considering multiple SNPs per gene.
**Table S13** Full GO enrichment analysis results considering one SNP per gene.
**Table S14** Summary of reduction and visualization of GO terms according to semantic similarity. It follows the genes with candidate adaptive SNPs on five‐ and three‐prime regions selected for discussion.
**Table S15** Pseudo *p*‐values of global and local measures of spatial autocorrelation for the genotypes significantly associated with strict winter conditions.
**Table S16** Pseudo *p*‐values of global and local measures of spatial autocorrelation for the selected bioclimatic variables representing winter conditions.

**Data S1** Supplemental methods, results, and discussion on the evaluation of alternative paralog filtering methods and the analysis of spatial autocorrelation in candidate adaptive SNPs and environmental variables.

## Data Availability

The demultiplexed Illumina sequence data (HiSeq 2000, single‐end reads of 100 bp) from 243 individual trees have been deposited in the European Nucleotide Archive (ENA) at EMBL‐EBI under accession number PRJEB71740 (https://www.ebi.ac.uk/ena/data/view/PRJEB71740).
