# Supplementary material for: Biological Processes Underlying Genetic Adaptation of Larches to Cold and Dry Winter Conditions in Eastern Siberia
Source: Ecol Evol. 2025 Feb 12;15(2):e70940. doi: 10.1002/ece3.70940 (PMC11821550; doi:10.1002/ece3.70940)
Supplement: Supplementary file 1 — Figure S1 Distribution of historical climate data. Seasonal (a) and annual (b) distribution of the median precipitation (mm), and minimum and maximum temperature (°C) measured at the sampling locations. Figure S2 Correlation analysis and hierarchical clustering among bioclimatic variables (Bio1–Bio19) and geographical coordinates. Only significant correlations (p < 0.01) are represented. Descriptions of the bioclimatic variables are in Table S5. Figure S3 Genotype pruning histograms and population structure assessed by PCA in R.SamBada. (a) Histogram of missingness with the vertical red line representing the threshold value of 0.2. (b) Histogram of minor allele frequency with the vertical red line representing the threshold value of 0.05. (c) Variance proportion of the first 100 PCA axes representing population structure with the vertical red line indicating the first PCA component included in R.SamBada multivariate analysis. Figure S4 Reduction and visualization of GO Terms according to semantic similarity. Treemap produced by REVIGO for biological processes considering multiple SNPs per gene (a) and one SNP per gene (b). Each rectangle represents a significantly (p < 0.05) over‐represented GO term. The size of each rectangle is proportional to the GO enrichment analysis based on Log10(p‐value) for that category. The four colors represent the four summarized GO term clusters represented in Figure 4, respectively; GO terms represented in white were not included in the summary. Detailed results are presented in Table S14. Figure S5 Cross‐validation procedure. Plot displaying the results of ADMIXTURE cross‐validation error considering 5 possible clusters. Figure S6 Admixture analysis. The proportion of admixtures based on two clusters (K = 2) were plotted along the longitude values of each individual location. The IDs of the individual tree were colored according to the assigned species taxonomy (L. gmelinii in blue, L. cajanderi in black). Figure S7 Genotypes’ distri [file ECE3-15-e70940-s001.docx]

**Supplemental Figures**

**
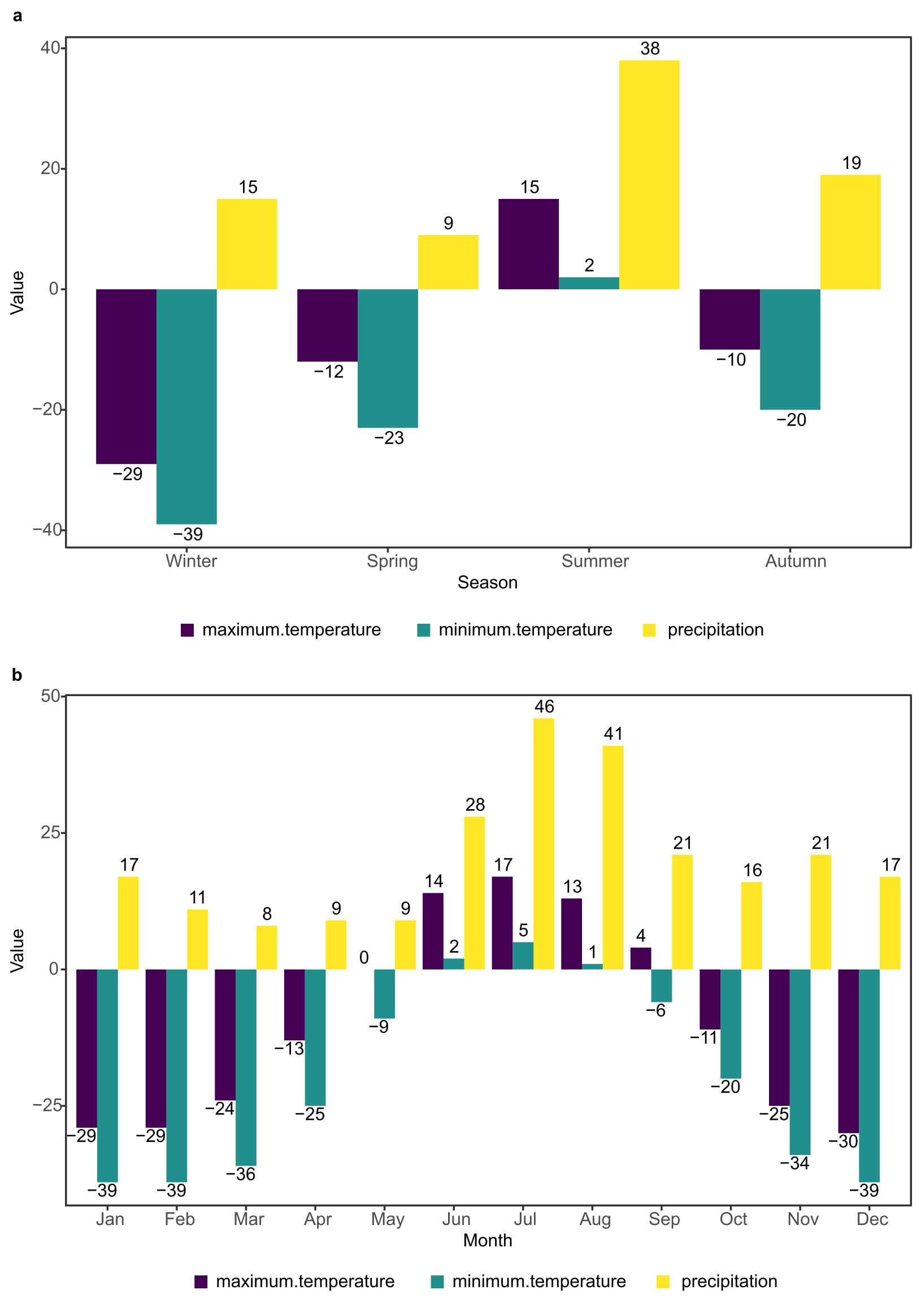
**

**Figure S1** Distribution of historical climate data. Seasonal (**a**) and annual (**b**) distribution of the median precipitation (mm), and minimum and maximum temperature (°C) measured at the sampling locations.


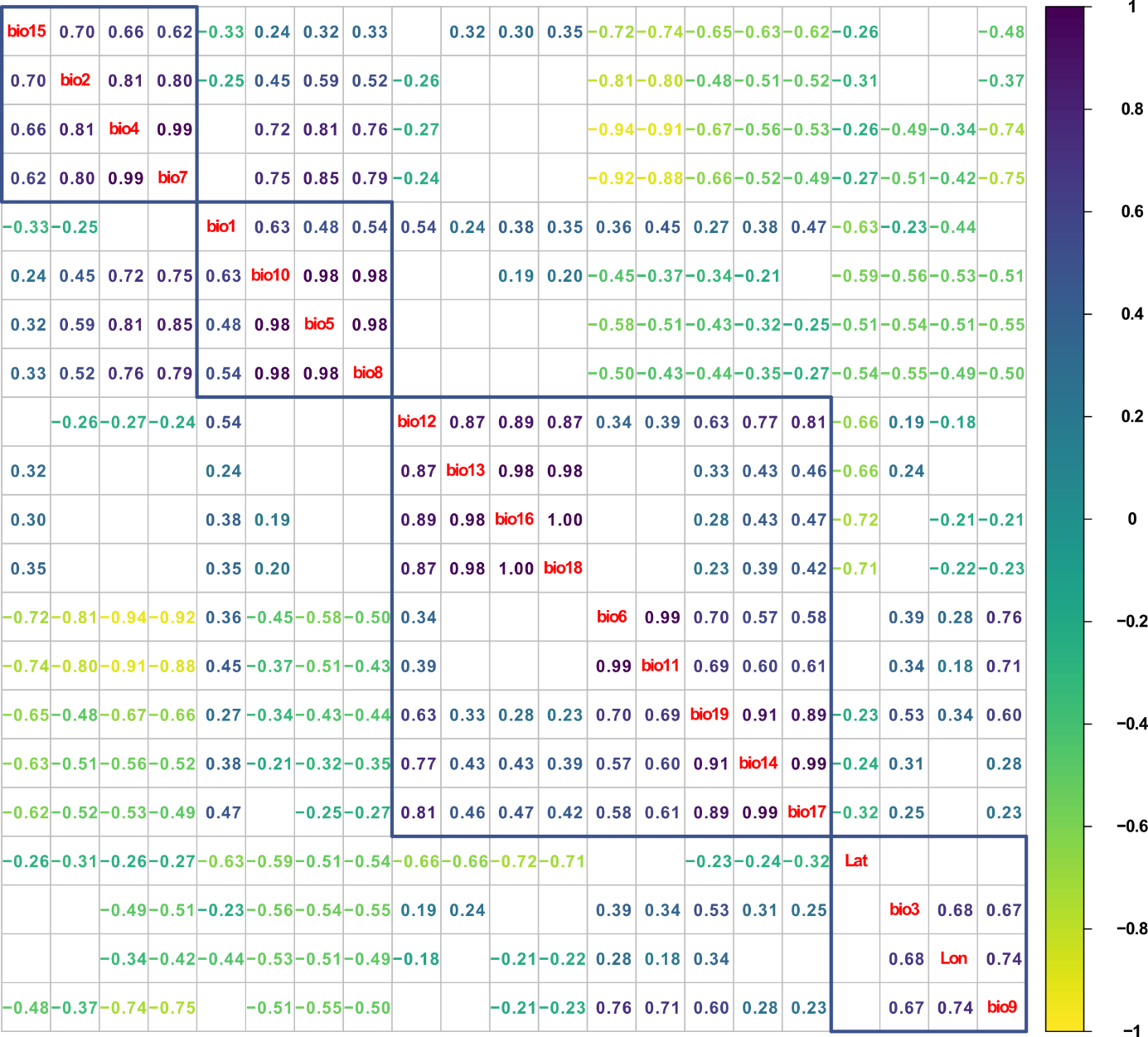


**Figure S2** Correlation analysis and hierarchical clustering among bioclimatic variables (Bio1–Bio19) and geographical coordinates. Only significant correlations (p<0.01) are represented. Descriptions of the bioclimatic variables are in **Table S5**.

**
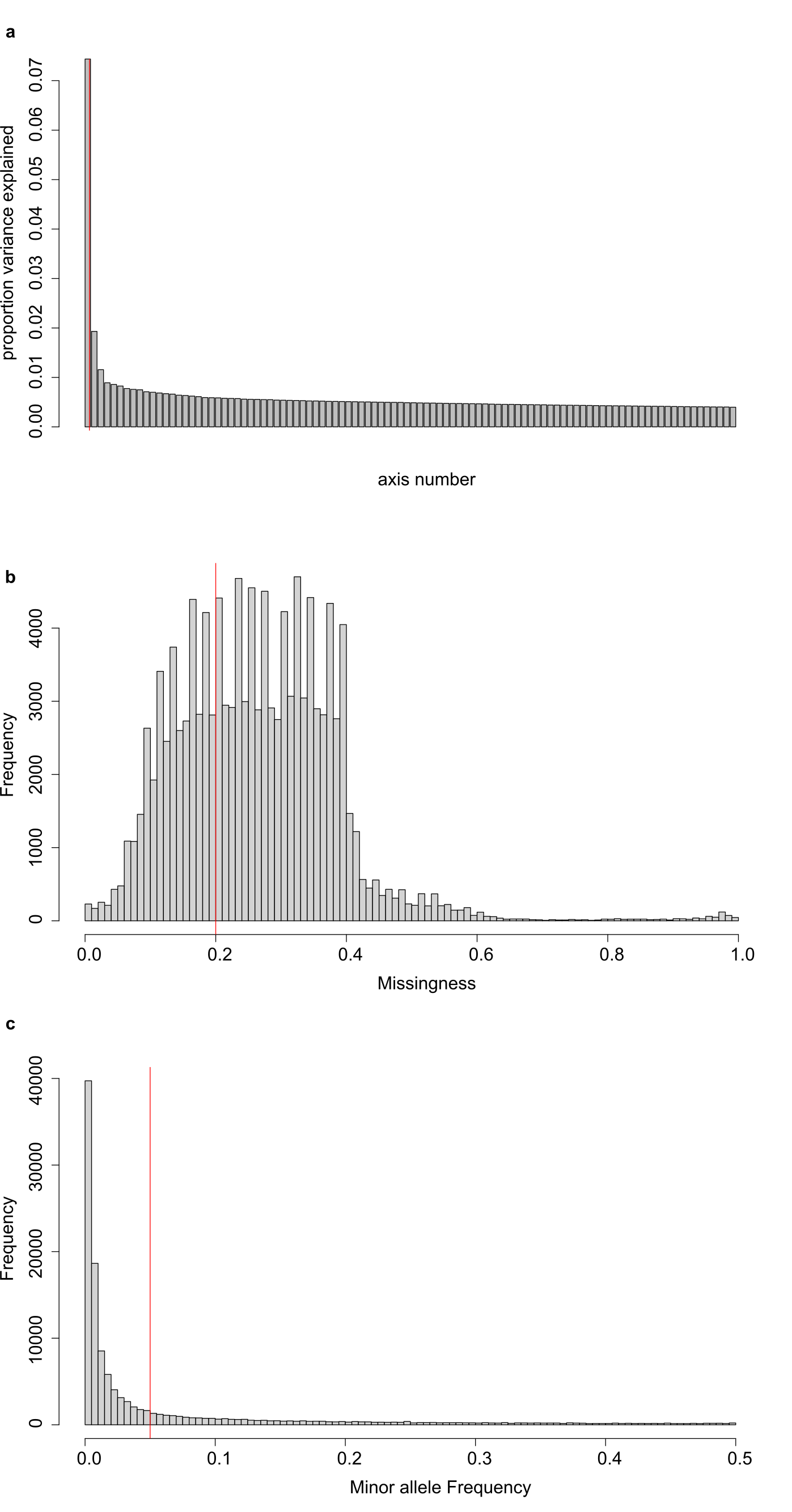
**

**Figure S3** Genotype pruning histograms and population structure assessed by PCA in R.SamBada. (**a**) Histogram of missingness with the vertical red line representing the threshold value of 0.2. (**b**) Histogram of minor allele frequency with the vertical red line representing the threshold value of 0.05. (**c**) Variance proportion of the first 100 PCA axes representing population structure with the vertical red line indicating the first PCA component included in R.SamBada multivariate analysis.

**
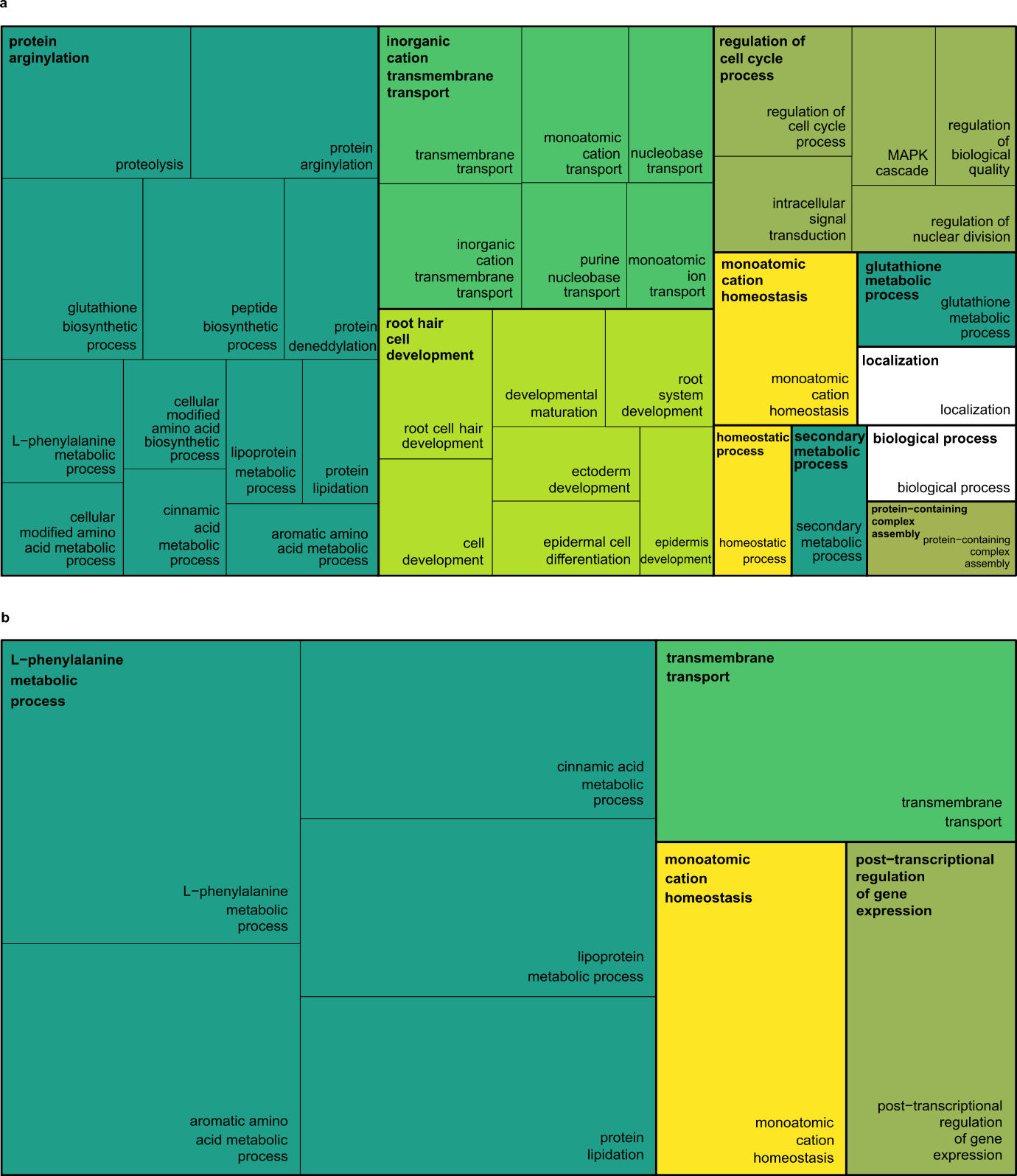
**

**Figure S4** Reduction and visualization of GO Terms according to semantic similarity. Treemap produced by REVIGO for biological processes considering multiple SNPs per gene (**a**) and one SNP per gene (**b**). Each rectangle represents a significantly (*p* < 0.05) over-represented GO term. The size of each rectangle is proportional to the GO enrichment analysis based on Log10(*p*-value) for that category. The four colors represent the four summarized GO term clusters represented in **Figure 4**, respectively; GO terms represented in white were not included in the summary. Detailed results are presented in **Table S14**.


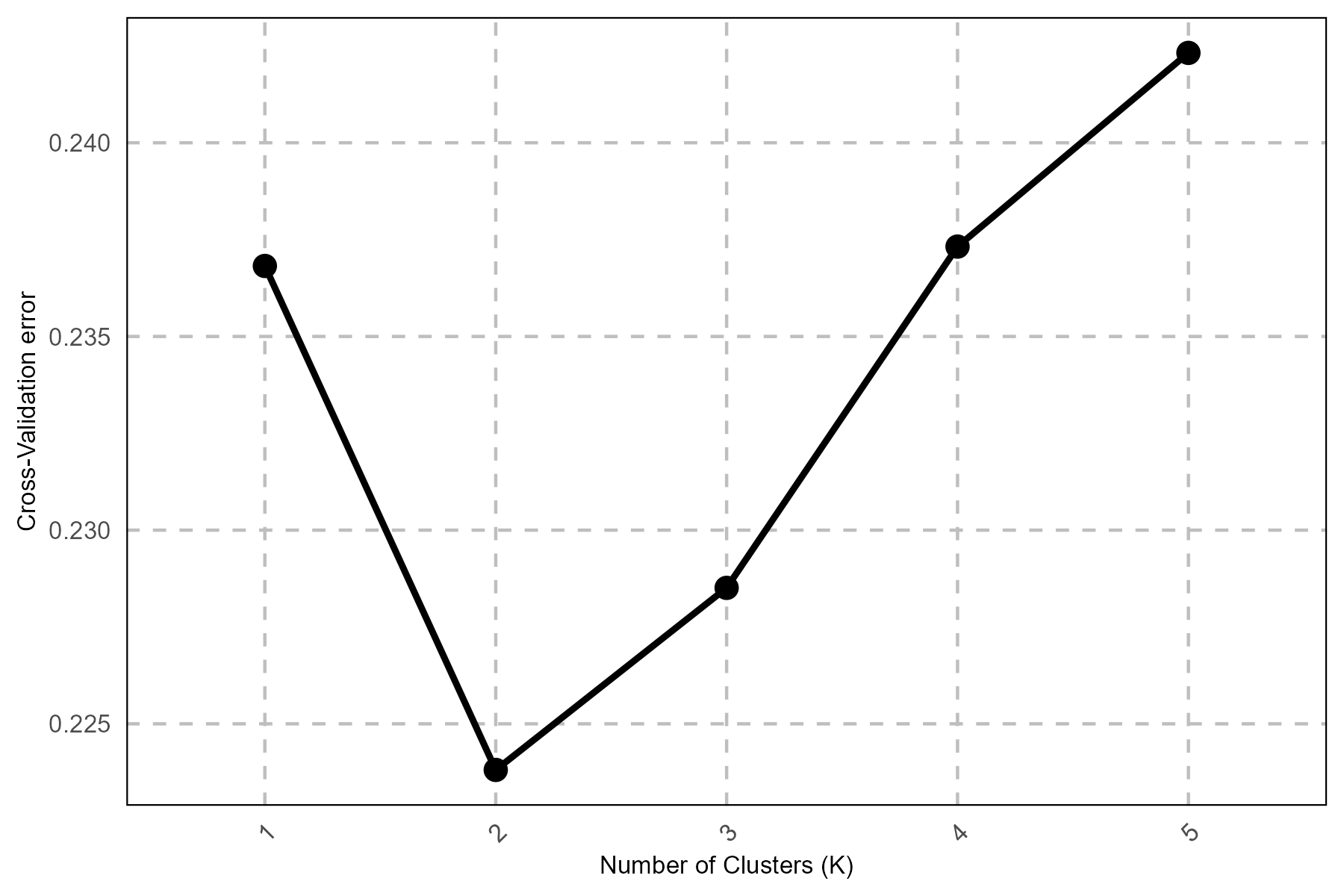


**Figure S5** Cross-validation procedure. Plot displaying the results of ADMIXTURE cross-validation error considering 5 possible clusters.


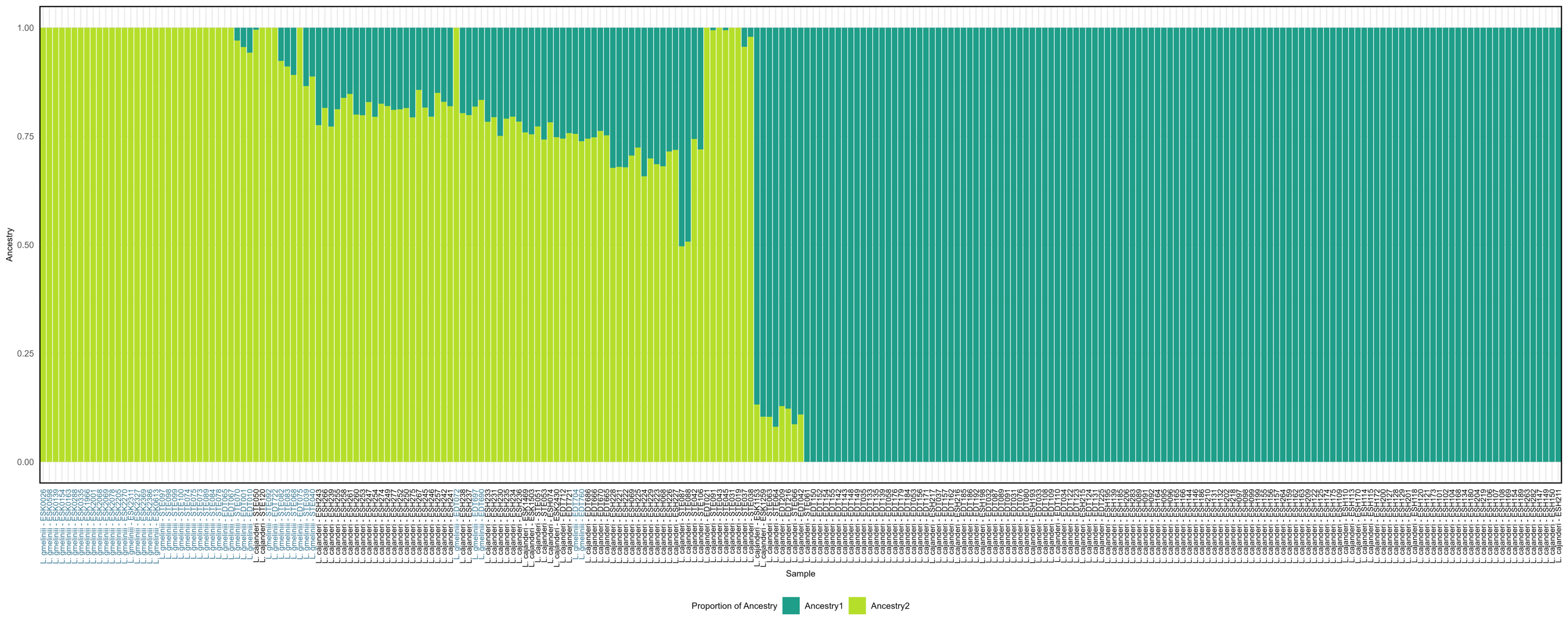
**Figure S6** Admixture analysis. The proportion of admixtures based on two clusters (*K* = 2) were plotted along the longitude values of each individual location. The IDs of the individual tree were colored according to the assigned species taxonomy (*L*. *gmelinii* in blue, *L*. *cajanderi* in black).


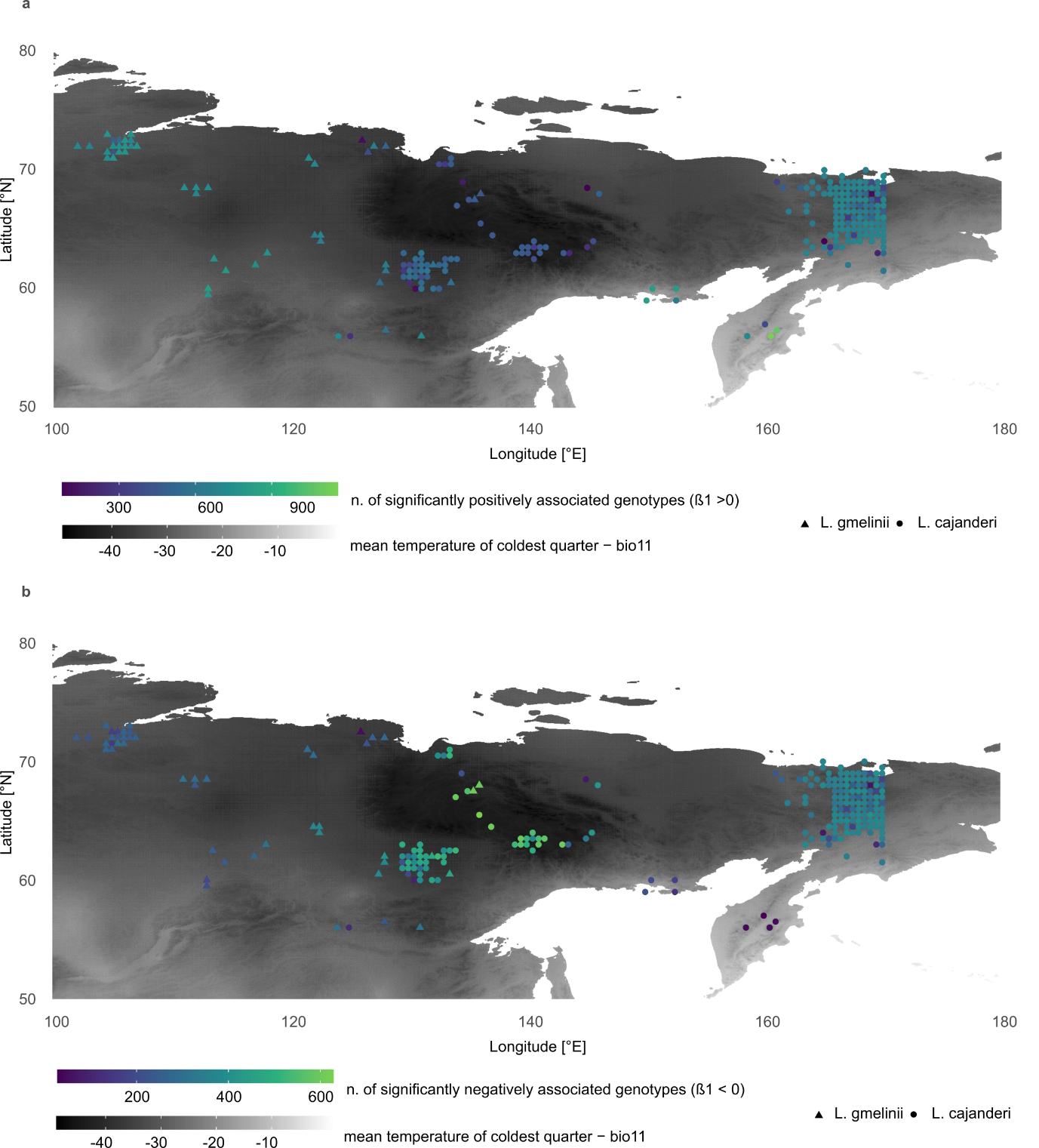


**Figure S7** Genotypes’ distribution. Map of the study area with background black-white shade representing the WorldClim bioclimatic variable Mean Temperature of Coldest Quarter (Bio11). Dots represent the individual sampling sites. Color dots represent the number of significantly associated genotypes with the group of four selected variables representing winter condition with (**a**) positive regression coefficient (*β1* > 0), and (**b**) negative regression coefficient (*β1* < 0). Individuals were spread on the map to allow visibility of all samples with color reflecting the number of genotypes.


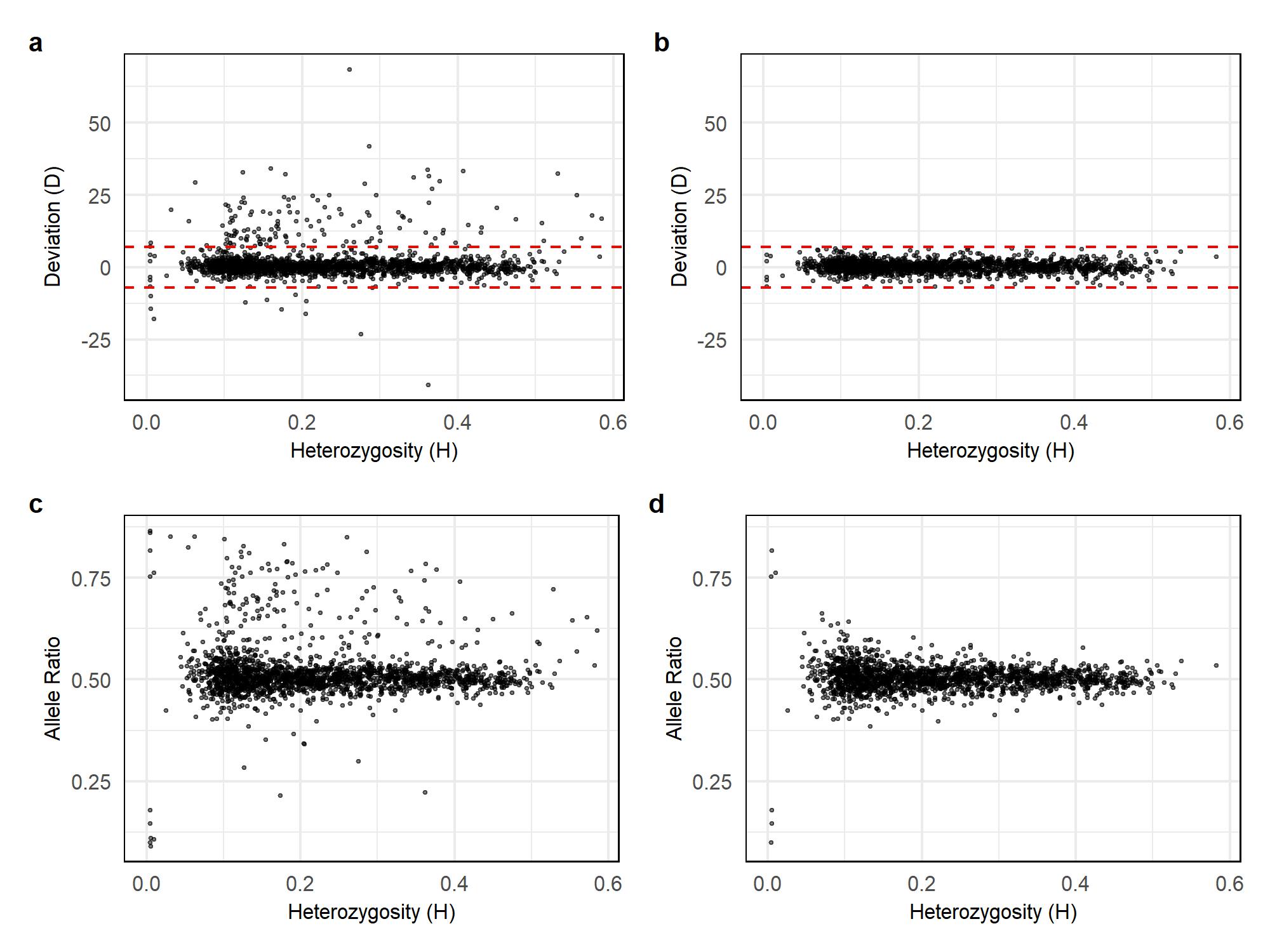


**Figure S8** Scatterplots visualizing the relationships between heterozygosity (H), deviation (D), and allele ratio before (using Stacks maximum mismatch threshold of 3) and after applying HDplot filtering thresholds. Panel (**a**) shows the distribution of loci based on H and D prior to filtering, with dashed red lines indicating the thresholds for deviation (|D| ≤ 7). Panel (**b**) presents the retained loci after filtering. Panels (**c**) and (**d**) illustrate the relationship between H and allele ratio before and after filtering, respectively.
