# Supplementary material for: Biological Processes Underlying Genetic Adaptation of Larches to Cold and Dry Winter Conditions in Eastern Siberia
Source: Ecol Evol. 2025 Feb 12;15(2):e70940. doi: 10.1002/ece3.70940 (PMC11821550; doi:10.1002/ece3.70940)
Supplement: Supplementary file 3 — Data S1 Supplemental methods, results, and discussion on the evaluation of alternative paralog filtering methods and the analysis of spatial autocorrelation in candidate adaptive SNPs and environmental variables. [file ECE3-15-e70940-s002.docx]

**Supplemental Information**

**Supplemental Material and Methods**

To compare alternative approaches for filtering potential paralogs, we evaluated the heterozygosity-based threshold (*max_shared_Hs_locus* = 0.6) used in our ipyrad pipeline against the HDplot method, which combines heterozygote proportions with deviations in allele read ratios (McKinney et al., 2017). Since the ipyrad pipeline generates VCF files lacking the Allelic Depth (AD) field required by HDplot for calculating allele ratios and deviations, we performed a de novo assembly using the Stacks pipeline to create a compatible VCF file (Catchen et al., 2013). Key parameters were aligned between the Stacks/HDplot and ipyrad/R.SamBada pipelines to ensure a meaningful comparison. In Stacks, we evaluated maximum mismatch thresholds of 2, 3, and 4 (*-M* 2, 3, and 4) for clustering to approximate the 90% similarity threshold (*clust_threshold* = 0.90) used in ipyrad. Missing data thresholds retained loci present in at least 80% of samples (*-r* 0.8), comparable to ipyrad’s filter retaining ~60% of samples (*min_samples_locus* = 146) and R.SamBada’s pruning, which excluded SNPs with >20% missing data. SNP density was restricted to a maximum of 22 SNPs per locus (*max-SNPs-per-locus =* 22), mirroring ipyrad’s parameter (*max_SNPs_locus* = 22). MAF filtering (*min-maf* = 0.05) excluded SNPs with a frequency below 5%, consistent with R.SamBada’s threshold (MAF = 0.05). Heterozygosity filtering in Stacks was implemented using the *max-obs-het* = 0.6 parameter, similar to ipyrad’s filters (*max_Hs_consens* = 0.05 and *max_shared_Hs_locus* = 0.6). Finally, the Stacks-derived VCF file was processed with HDplot, applying thresholds for heterozygosity (H ≤ 0.6) and deviation (|D| ≤ 7), as recommended by McKinney et al. (2017), to exclude loci with imbalanced allele read ratios. Deviation (D) and allele ratio plots were generated using the package ggplot2 (Wickham, 2016).

**Supplemental Results and Discussion**.

To compare alternative approaches for filtering potential paralogs, we evaluated the HDplot method, which integrates heterozygote proportions with deviations in allele read ratios (McKinney et al., 2017). In contrast to the ipyrad pipeline which yielded 8,604 SNPs after filtering, the Stacks pipeline, with maximum mismatch thresholds of 2, 3, and 4 (-M 2, 3, and 4), produced 1,889, 1,942, and 1,944 SNPs, respectively. Applying the HDplot deviation filter (|D| > 7) further reduced these datasets to 1,699, 1,767, and 1,764 SNPs, respectively, excluding approximately 9–10% of loci with deviations from the expected 1:1 allele read ratio for supposedly heterozygous loci (**Figure S8**). The application of HDplot, by excluding loci with significant deviations in allele read ratios, likely improved the removal of potential paralogs and sequencing artifacts. However, the combined stringency of the Stacks pipeline and HDplot filtering may also exclude SNPs with minor imbalances that could hold biological significance, and limit the statistical power to detect adaptive signals at the population level. Given the broader genomic representation retained by the ipyrad pipeline, we chose this approach for our study. The larger SNP dataset aligns more closely with our broader objective of investigating potential biological processes enriched by candidate adaptive SNPs underlying genetic adaptation.

To account for underlying patterns of spatial association due to population structure, spatial autocorrelation was measured using Moran’s *I* for the 1,288 genotypes significantly associated with the group of four selected variables with negative regression coefficient (*β1* < 0) as well as for the environmental variables with a weighting scheme of 20 neighbors. Pseudo *p*-values for the measures of spatial autocorrelation are presented in **Table S15 and S16**. A total of 51.2% of the genotypes showed significant (*p* < 0.02) global measures of spatial autocorrelation. An average of 22.1% of genotypes in each individual tree showed significant (*p* < 0.02) local Moran´s *I* positive values, indicating the tendency of closely related trees to cluster. All four environmental variables show significant global Moran´s *I* values, which possibly contribute to the significant spatial autocorrelation in candidate adaptive SNPs. Although spatial dependency in neutral polymorphic markers might increase the probability of detecting them as potentially under selection, it is also seen that spatial autocorrelation of true candidate adaptive SNPs increases with selection pressure (Stucki et al., 2017).
